# Dialysis Patients with Implanted Drug-Eluting Stents Have Lower Major Cardiac Events and Mortality than Those with Implanted Bare-Metal Stents: A Taiwanese Nationwide Cohort Study

**DOI:** 10.1371/journal.pone.0146343

**Published:** 2016-01-05

**Authors:** Hsin-Fu Lee, Lung-Sheng Wu, Yi-Hsin Chan, Cheng-Hung Lee, Jia-Rou Liu, Hui-Tzu Tu, Ming-Shien Wen, Chi-Tai Kuo, Wei-Jan Chen, Yung-Hsin Yeh, Lai-Chu See, Shang-Hung Chang

**Affiliations:** 1 Chang Gung University and Department of Cardiology, Chang Gung Memorial Hospital, Taipei, Taiwan; 2 Department of Public Health, College of Medicine, Chang Gung University, Taoyuan, Taiwan; 3 Biostatistics Core Laboratory, Molecular Medicine Research Center, Chang Gung University, Taoyuan, Taiwan; Osaka University Graduate School of Medicine, JAPAN

## Abstract

**Objective:**

To investigate the efficacy and long-term clinical benefits of DES for dialysis patients.

**Background:**

It is unclear whether percutaneous coronary intervention (PCI) with drug-eluting stent (DES) implantation is associated with lower rates of major adverse cardiovascular events (MACE) or mortality compared to bare-metal stents (BMS).

**Methods:**

From a nationwide cohort selected from Taiwan’s National Health Insurance Research Database, we enrolled 2,835 dialysis patients who were hospitalized for PCI treatment with stent implantation from Dec 1, 2006. Follow-up was from the date of index hospitalization for PCI until the first MACE, date of death, or December 31, 2011, whichever came first.

**Results:**

A total of 738 patients (26.0%) had DES implanted, and 2,097 (74%) had BMS implanted. The medium time to the first MACE was 0.53 years (interquartile range: 0.89 years; range: 0–4.62 years). At 1-year follow-up, patients treated with BMS had significantly, non-fatal myocardial infarction (MI), all-cause mortality, and composite MACE compared to those treated with DES. The overall repeat revascularization with coronary artery bypass graft (CABG), non-fatal MI, all-cause mortality, and composite MACE were significantly lower in patients treated with DES than those treated with BMS. Multivariate cox regression analysis showed that older age, history of diabetes, history of heart failure, history of stroke, and DES vs. BMS were independent significant predictors of MACE.

**Conclusions:**

DES implantation conferred survival benefits in dialysis patients compared with BMS implantation.

## Introduction

Coronary artery disease (CAD) is prevalent in more than 50% of patients with end-stage renal disease (ESRD) who are on hemodialysis [1]. The presence of complex lesions such as massive calcification of coronary lesions, and multi-vessel disease increases the risk of death in these patients compared with non-dialysis patients [2–5]. Coronary angioplasty with stenting has been proven to be an effective treatment for CAD in the ESRD population. However, the use of coronary stents in dialysis patients is still under debate because this population has been consistently excluded from large studies which compared the efficacy of drug-eluting stents (DES) to bare-metal stents (BMS) [6–9]. The increased levels of coronary calcification and arterial stiffness in the ESRD population may increase the severity of vascular injury after either angioplasty or stenting, predisposing them to restenosis [10, 11]. Furthermore, dialysis is associated with the activation of the coagulation system, increased platelet aggregation [12], induced inflammatory response [13], and the release of oxidant free radicals [14], which may contribute to neointimal hyperplasia. The anti-proliferation effect of DES should be useful in this situation. However, calcification could diminish the biological effects of anti-proliferative drugs due to suboptimal drug delivery and absorption [15], so calcification may attenuate the efficacy of DES. Data for DES use in dialysis patients are scarce, observational in nature, and based on retrospective analyses of small cohorts [16–23]. Additionally, these studies are too weak to offer dependable conclusions. We therefore conducted this nationwide dynamic cohort study using data from the Taiwan National Health Insurance Research Database (NHIRD) to investigate the efficacy and long-term clinical benefits of DES for dialysis patients.

## Methods

### Data source

The National Health Insurance (NHI) program in Taiwan provides compulsory universal health insurance to 98% of the population (22.6 million of a total of 23 million people). The program, which was implemented in 1995, covers all forms of health care services. The National Health Insurance Administration (NHIA) and the National Health Research Institute (NHRI) jointly manage and maintain all insurance claim data in the NHIRDs. The NHIRDs contain comprehensive information for all enrollees, including birth date, gender, diagnostic codes, surgery or procedures received, medications prescribed, admission date, hospitalization, discharge date, medical institutions codes, and claim amounts. Routine auditing of claims by the NHIA helps ensure the accuracy and validity of NHIRD data [24–26]. Disease diagnoses are coded according to the *International Classification of Disease*, *Ninth Revision*, *Clinical Modification* (ICD-9-CM). The diagnosis of ESRD was assigned to chronic kidney disease patients with dialysis. In Taiwan, patients with ESRD are eligible for a catastrophic illness certificate after being reviewed by two specialists based on clinical presentations and laboratory studies. Percutaneous coronary intervention (PCI) is reimbursed by the NHI, and the NHIRD maintains a record of patients with CAD who receive PCI with BMS or DES implants. Note that the first DES was reimbursed by Taiwan NHI in Dec 2006.We conducted a secondary data analysis using the NHIRDs and restricted the study period to between December 1, 2006, and December 31, 2011. This study was approved by the Ethics Institutional Review Board of Chang Gung Memorial Hospital. Informed consent was waived because the identification number of each patient had already been encrypted for privacy protection.

### Study Design

**Fig 1:**
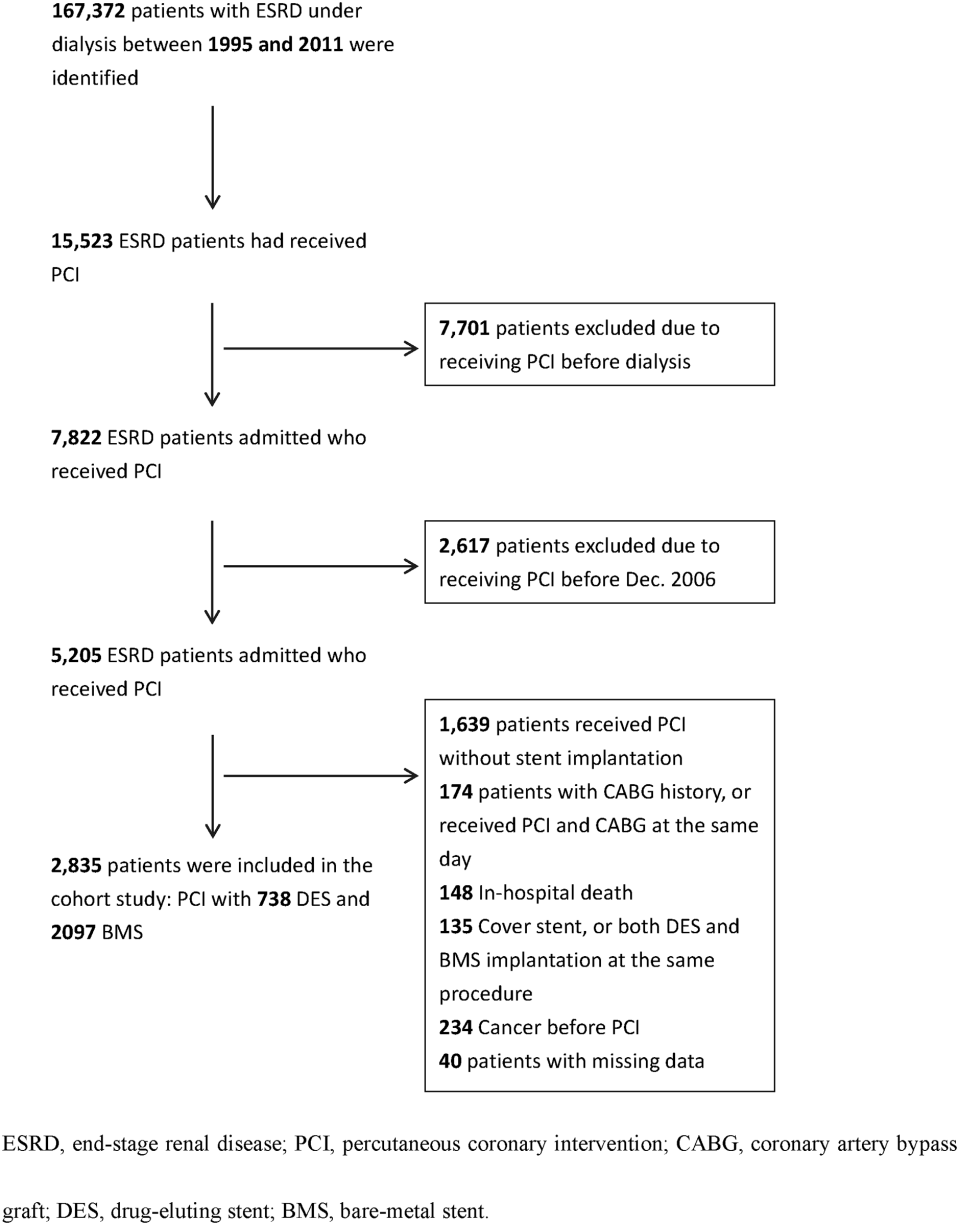
Flow chart of enrollment.

We used a nationwide cohort of Taiwanese patients who had been diagnosed with ESRD between December 1, 2006 and December 31, 2011. All enrolled patients were admitted for PCI, and followed up until 2011. Patients were excluded if 1) they had received PCI without stent implantation, 2) they had previously received coronary artery bypass graft (CABG) surgery, or they had received PCI and CABG on the same day, 3) died while hospitalized, 4) had DES as well as BMS implantations during the same procedure, 5) had been diagnosed with cancer before they received PCI, or 6) their diagnosis, PCI, or medication data were missing (Fig 1).

### Outcomes and covariate measurements

Variables associated with adverse outcome for patients undergoing PCI were selected for analysis [27, 28]. Co-morbidities at the time of PCI were identified by ICD-9-CM diagnosis codes and medication during index hospitalization. The following outcomes were major adverse cardiovascular events (MACEs): 1) all-cause mortality, 2) hospitalization and principal diagnosis of myocardial infarction (MI) (ICD-9-CM code410.x), 3) repeat revascularization for PCI or CABG, and 4) hospitalization and principal diagnosis of stroke (ICD-9-CM code 430–437). Subjects were followed up from the date of index hospitalization for PCI to the date of the first MACE, date of death, or December 31, 2011, whichever came first.

### Statistical analysis

The chi-square test or unpaired t-test was used to compare data between the DES and BMS study groups in the univariate analysis, where appropriate. The event-free rates for the first events were calculated using the Kaplan-Meier method. We used Cox proportional hazards models to assess univariate and multivariable covariates associated with progression of all patients to the endpoints. All prognostic variables with P<0.05 in univariate analysis and forward selection was used to determine the final Cox’s model to determine independent predictors. Hazard ratios (HRs) and their 95% confidence intervals (CIs) were computed. The assumption of proportional hazards was validated by plotting ln [-ln (survival function)] against ln (follow-up time). The two lines in this plot are parallel, validating the assumption of proportional hazards (result not shown). The significance level of this study was 0.05.

## Result

A total of 2,835 dialysis patients who had PCI treatment with stent implantation were eligible for this study (Fig 1). The mean age (±standard deviation) of the patients was 64.5±11.0 years, and the study population included 58.3% males. The mean duration from dialysis to the first PCI was 3.72±3.25 years. About one quarter of the subjects (n = 738, 26.0%) received DES, and 2,097 (74%) received BMS. The DES group had a significantly higher prevalence of dyslipidemia and use of statins compared to the BMS group (82.7% vs. 76.1%, P = 0.0002; 34.8% vs. 29.8%, P = 0.0105, respectively). However, the BMS group had a significantly higher frequency of strokes compared to the DES group (43.0% vs. 37.9%, P = 0.0162). The DES group had a slightly but significantly lower number of stent implantations than the BMS group (1.1 ± 0.4 vs. 1.2 ± 0.5, P< 0.0001). There was no significant difference in age, sex, hypertension, diabetes mellitus, atrial fibrillation, congestive heart failure, acute coronary syndrome (ACS), previous gastrointestinal (GI) bleeding, and prescribed medication between the two study groups (Table 1).

**Table 1:** Patient demographics, co-morbidities, and stent characteristics at stent implantation among dialysis patients.

	Total	DES group	BMS group	P value
**Number of patient**	2,835	738	2,097	
**Demographic**				
Age, years	64.5±11.0	64.9 ± 10.7	64.4± 11.1	0.2340^1^/0.1939^2^
0–49, n (%)	253 (8.9)	50 (6.8)	203 (9.7)	
50–64, n (%)	1,160 (40.9)	311 (42.1)	849 (40.5)	
65–74, n (%)	833 (29.4)	217 (29.4)	616 (29.4)	
75–84, n (%)	514 (18.1)	141 (19.1)	373 (17.8)	
85+, n (%)	75 (2.7)	19 (2.6)	56 (2.7)	
Male, n (%)	1,652 (58.3)	446 (60.4)	1,206 (57.5)	0.1661^2^
**Co-morbidities**				
Hypertension, n (%)	2,784 (98.2)	727 (98.5)	2,057 (98.1)	0.4636^2^
DM, n(%)	2,275 (80.3)	599 (81.1)	1,676 (79.9)	0.4662^2^
Dyslipidemia, n (%)	2,205 (77.8)	610 (82.7)	1,595 (76.1)	0.0002^2^
CHF, n (%)	1,461 (51.5)	362 (49.1)	1,099 (52.4)	0.1166^2^
Previous Stroke, n (%)	1,182 (41.7)	280 (37.9)	902 (43.0)	0.0162^2^
ACS, n (%)	773 (27.3)	190 (25.8)	583 (27.8)	0.2806^2^
Atrial fibrillation, n (%)	203 (7.2)	48 (6.5)	155 (7.4)	0.4213^2^
Previous GI bleeding, n (%)	115 (4.1)	28 (3.8)	87 (4.2)	0.6744^2^
**Medication**				
Aspirin, n (%)	2,333 (82.3)	620 (84.0)	1,713 (81.7)	0.1551^2^
Clopidogrel, n (%)	2,793 (98.2)	729 (98.8)	2,064 (98.4)	0.4934^2^
ACEI/ARB, n (%)	1,348 (47.6)	367 (49.7)	981 (46.8)	0.1678^2^
Beta-blocker, n (%)	1,038 (36.6)	276 (37.4)	762 (36.3)	0.6069^2^
Statin, n (%)	881 (30.1)	257 (34.8)	624 (29.8)	0.0105^2^
**Stent Implantation**				
No. of vessels intervened				0.0190^3^
1 vessel, n (%)	1,867 (65.6)	465 (63.0)	1,402 (66.9)	
2 vessels, n (%)	849 (30.0)	232 (31.4)	617 (29.4)	
3 vessels, n (%)	119 (4.2)	41 (5.6)	78 (3.7)	
Number of stents implanted				<0.0001^3^
1 stent, n (%)	2,365 (83.4)	662 (89.7)	1,703 (81.2)	
2 stents, n (%)	394 (13.9)	66 (8.9)	328 (15.6)	
3 stents, n (%)	68 (2.4)	7 (1.0)	61 (2.9)	
4 stents, n (%)	8 (0.3)	3 (0.4)	5 (0.2)	

P values were calculated with the use of ^1^independent t test, ^2^chi-square test and ^3^chi-square test for trend.

Values are expressed as mean ± standard deviation or number (percentage).

DES, drug-eluting stent; BMS, bare-metal stent; ACS, acute coronary syndrome; DM, diabetes mellitus; CHF, congestive heart failure; GI, gastrointestinal; ACEI, angiotensin converting enzyme inhibitor; ARB, angiotensin receptor blocker.

**Fig 2:**
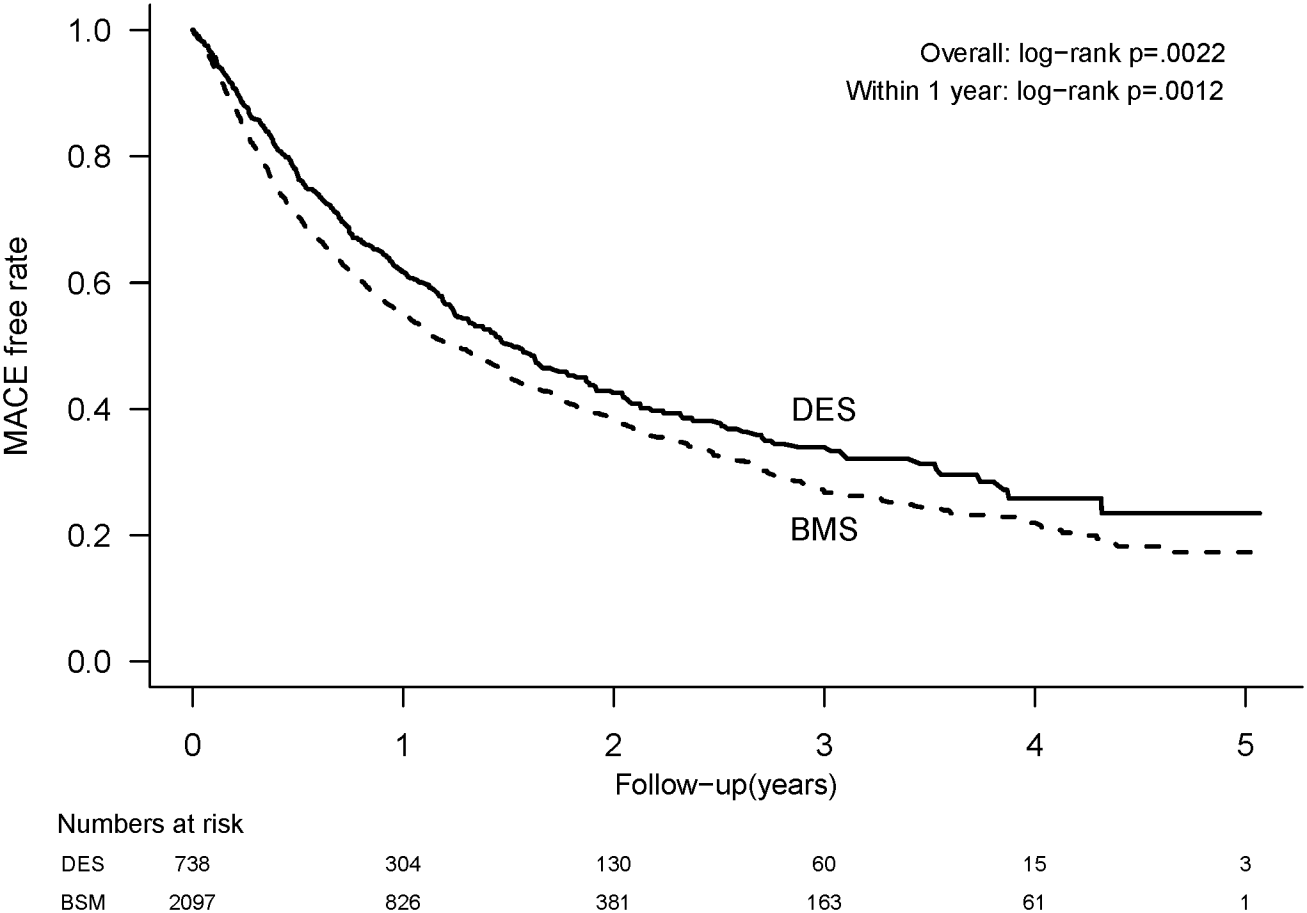
MACE-free rates among dialysis patients after implantation of drug-eluting stents (DES) or bare-metal stents (BMS).

**Fig 3:**
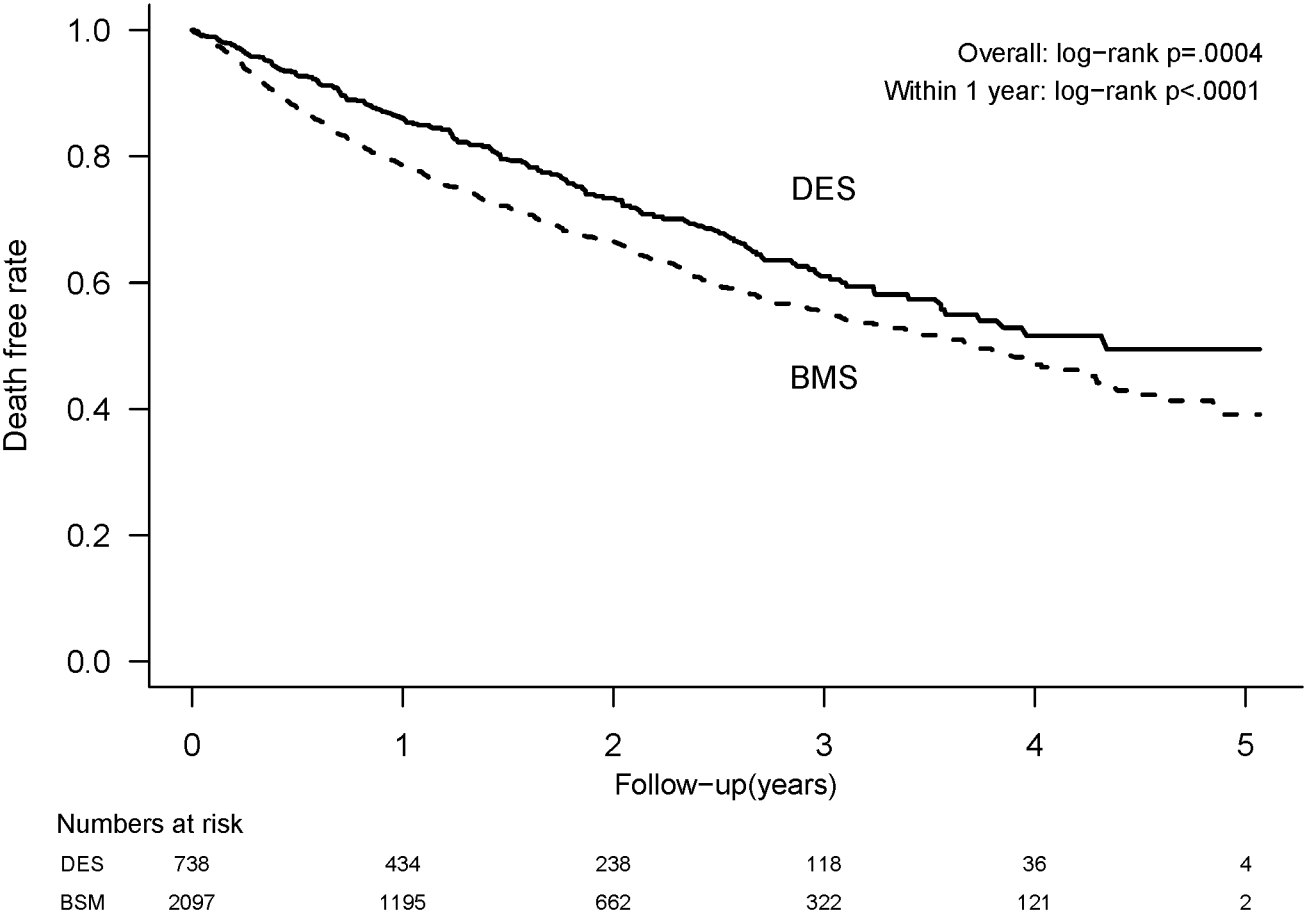
Survival rates among dialysis patients after implantation of drug-eluting stents (DES) or bare-metal stents (BMS).

The mean follow-up duration was 2.06 ± 0.08 years in the DES group and 1.89 ± 0.04 years in the BMS group. The mean time to the first cardiac event was 0.53 year (interquartile range: 0.89 years; range: 0–4.62 years). At the 1-year follow-up, patients who received BMS had significantly higher non-fatal MI (6.1% vs. 3.8%, P = 0.0177), all-cause mortality (12.6% vs. 8.7%, P<0.0001), and composite MACE (40.2% vs. 32.5%, P = 0.0012) compared to those treated with DES. In addition, the BMS group had a significantly higher overall repeat revascularization with CABG (1.6% vs. 0.5%, P = 0.0277), non-fatal MI (7.5% vs. 5.0%, P = 0.0266), all-cause mortality (19.3% vs. 13.8%, P = 0.0004), and composite MACE (55.8% vs. 47.2%, P = 0.0022) compared to the DES group (Table 2). Kaplan-Meier curves were plotted and showed a lower rate of MACE and all-cause mortality in the DES group than the BMS group (Figs 2 and 3).

**Table 2:** One year and overall major adverse cardiac events (MACEs) after stent implantation.

	Total	DES group	BMS group	P value
Number of patient	2,835	738	2,097	
One-Year MACE, n (%)	1,082 (38.2)	240 (32.5)	842 (40.2)	0.0012
Death from any cause	328 (11.6)	64 (8.7)	344 (12.6)	<0.0001
Non-fatal MI	155 (5.5)	28 (3.8)	127 (6.1)	0.0177
Repeat revascularization-PCI	488 (17.2)	125 (16.9)	363 (17.3)	0.6446
Repeat revascularization-CABG	24 (0.9)	3 (0.4)	21 (1.0)	0.1213
Stroke	87 (3.1)	20 (2.7)	67 (3.2)	0.4584
Overall MACE, n (%)	1,519(53.6)	348(47.2)	1,171(55.8)	0.0022
Incidence per 100 person-years	48.23	42.06	50.43	
Death from any cause	506 (17.9)	102 (13.8)	404 (19.3)	0.0004
Non-fatal MI	194 (6.8)	37 (5.0)	157 (7.5)	0.0226
Repeat revascularization-PCI	651 (23.0)	175 (23.7)	476 (22.7)	0.7965
Repeat revascularization-CABG	38 (1.3)	4 (0.5)	34 (1.6)	0.0277
Stroke	130 (4.6)	30 (4.1)	100 (4.8)	0.3944

P values was calculated with the use of log-rank test.

Values are expressed as number (percentage).

MI, myocardial infarction; PCI, percutaneous coronary intervention; CABG, coronary artery bypass graft.

Univariate analysis showed that older age, history of diabetes, history of heart failure, history of stroke, ACS, and DES vs. BMS were significant predictors of MACE. After adjusting for confounders, multivariate analysis showed that older age, history of diabetes (HR = 1.296, 95% CI = 1.134–1.482), history of heart failure (HR = 1.231, 95% CI = 1.112–1.364), history of stroke (HR = 1.118, 95% CI = 1.007–1.240), ACS (HR = 1.333, 95% CI = 1.194–1.489), and DES vs. BMS (HR = 0.837, 95% CI = 0.743–0.944) were independent significant predictors of MACE (Table 3).

**Table 3:** Univariate and multivariate analysis of major adverse cardiac events (MACEs) among dialysis patients after stent implantation.

	Univariate analysis		Multivariate analysis	
	HR (95% CI)	P value	HR (95% CI)	P value
Age, years				
20–49	0.868 (0.715–1.054)	0.1537	0.895 (0.736–1.087)	0.2632
50–64	reference group	-		
65–74	1.138 (1.008–1.284)	0.0366	1.095 (0.969–1.237)	0.1456
75–84	1.240 (1.077–1.428)	0.0028	1.186 (1.028–1.369)	0.0195
85+	1.654 (1.228–2.227)	0.0009	1.535 (1.138–2.071)	0.0050
Male	1.095 (0.987–1.221)	0.0865		
Hypertension	1.086 (0.752–1.569)	0.6585		
DM	1.302 (1.140–1.487)	< .0001	1.296 (1.134–1.482)	0.0001
Dyslipidemia	0.998 (0.885–1.127)	0.9791		
CHF	1.255 (1.134–1.388)	< .0001	1.231 (1.112–1.364)	< .0001
Previous Stroke	1.186 (1.072–1.312)	0.0010	1.115 (1.005–1.238)	0.0401
ACS	1.349 (1.208–1.506)	< .0001	1.333 (1.194–1.489)	< .0001
AF	1.146 (0.950–1.382)	0.1547		
Previous GI bleeding	0.963 (0.745–1.244)	0.7725		
Aspirin	0.941 (0.827–1.071)	0.3555		
Clopidogrel	0.863 (0.577–1.292)	0.4745		
ACEI/ARB	0.979 (0.885–1.083)	0.6826		
Beta-blocker	0.961 (0.866–1.067)	0.4569		
Statin	0.956 (0.856–1.066)	0.4160		
No. of vessels intervened				
1 vessel	reference group	-		
2 vessels	1.105 (0.991–1.233)	0.0726		
3 vessels	0.973 (0.753–1.258)	0.8364		
Number of stents implanted				
1 stent	reference group	-		
2 stents	1.092 (0.944–1.264)	0.2348		
3 stents	1.190 (0.858–1.650)	0.2982		
4 stents	0.769 (0.248–2.387)	0.6494		
DES versus BMS	0.830 (0.736–0.935)	0.0022	0.837 (0.743–0.944)	0.0038

HR, hazard ratio; CI, confidence interval; DM, diabetes mellitus; AF, atrial fibrillation; CHF, congestive heart failure; ACS, acute coronary syndrome; GI, gastrointestinal; ACEI, angiotensin converting enzyme inhibitor; ARB, angiotensin receptor blocker; DES, drug-eluting stent; BMS, bare-metal stent.

The duration of aspirin use was 190.6 ± 195.8 days in the DES group and 172.4 ± 199.5 days in the BMS group (P = 0.051). The duration of clopidogrel use was 166.7 ± 165.1 days in the DES group and 132.1 ± 150.2 days in the BMS group (P<0.001).GI bleeding rate after implantation of stent was 1.49% in the DES group and 1.96% in the BMS group (P = 0.4118).

## Discussion

To our knowledge, there are currently no data available from prior large-scale cohort studies or randomized controlled trials comparing the outcome of MACEs or mortality among dialysis patients following PCI with DES versus BMS. This study identified an association between a lower rate of MACE in dialysis patients in the DES group than those in the BMS group. This difference persisted after adjusting for important confounders. The superior efficacy of DES appears to be predominantly in the improvement of all-cause mortality.

The baseline characteristics of patients in the DES and BMS groups were obviously heterogeneous and it is possible that many of these might have an impact on the outcomes measured. For example, the number of stent implantations was slightly, but significantly higher in the BMS group. Stent implantation number was previously shown to be an independent predictor of restenosis and stent thrombosis [29, 30]. Our data showing that patients in the BMS group had a higher incidence of prior strokes, which could have been associated with higher MACE, were also consistent with previous findings [27, 28]. Although the percentage of ACS was not significantly different among the groups, ACS was a significant prognostic predictor for the MACE after stent implantation; which is compatible with a historical study [31]. In addition, patients with chronic kidney disease including dialysis population presenting with ACS were associated with increased risk of death and MI [32].

Although 12 months of dual antiplatelet therapies (DAPT) for ACS patients or after DES implantation were followed by current guidelines [33], the duration was according to the clinical judgment of physicians in the cohort study. Because a higher MACE rate contributing to early discontinuation of DAPT and individualized drug therapy in our study groups, the duration of DAPT was less than 12 months.

Bleeding complication has been well studied with antiplatelet treatment, especially with dual antiplatelet therapies. We analyzed GI bleeding which is one of the most common bleeding complications in patients receiving antiplatelet therapy [34, 35]. In addition, high risk of GI bleeding has been observed for dialysis patients [36], a longer duration of dual antiplatelet agents, which is commonly advised for DES implanted patients, may be associated with higher risk of GI bleeding. Although the DES group had a longer duration of using antiplatelet agents in our cohort, there was still no significant difference of GI bleeding after stent implantation between the DES and BMS groups. Higher MACE rates in our study groups leading to the under detection of possibly later end-point of GI bleeding, or the potential confounding of unmeasured factor like proton-pump inhibitor use may have interfered with the analysis.

Unlike other studies in which follow-up angiography was used to analyze the risk of target lesion or target vessel revascularization (TLR or TVR), our cohort came with data about ischemia-driven revascularization of PCI or CABG. Our data offered a realistic view of what is observed in the clinic. However, since silent myocardial ischemia may be prevalent in this population [1], our present study may have underestimated the efficacy of DES in reducing the risk of restenosis which was previously observed in other studies [18, 23]. This could be why our data did not show a significant difference in repeat revascularization for PCI between the DES and the BMS groups. However, patients in the DES group had a lower risk of repeat revascularization for CABG. This was possibly because patients with severe CAD who were also candidates for CABG underwent clinical or angiographic follow-ups in quick succession, which might have influenced clinicians’ judgment.

Our data, showing that DES use was associated with a reduction in myocardial infarction rates, were not supported by previously published studies [16–23]. MI was defined as hospitalization with a principal diagnosis of MI in the present study. However, other studies defined it as elevation of cardiac enzymes and/or the development of new pathological Q on electrocardiogram. Importantly, our data demonstrated that DES reduced hospitalization for MI. We believe that the definition might make a difference in the end-point measured.

This study echoes the findings of Halkin et al., reported in the National Heart, Lung and Blood Institute Dynamic Registry, that patients treated with DES had lower cumulative 1-year rates of all-cause mortality compared to those treated with BMS [22]. The mechanism underlying higher survival rates in the DES group remain unclear. Based on previous reports that acute myocardial infarction and all other cardiac causes accounted for the majority of mortality in dialysis patients [37], we hypothesized that higher vessel patency rates afforded by DES caused less damage to the myocardium, thereby leading to a lower tendency of fatal outcomes after episodes of acute ischemia and/or arrhythmia. Our findings may have several important clinical implications: 1) Although Halkinet al. first reported the survival benefit conferred by DESs compared with BMSs, they had only a small sample size with 74 subjects (BMS n = 41 and DES n = 33). A meta-analysis study done by Abdel-Latif et al. also found a trend towards lower all-cause mortality in DES versus BMS dialysis patients [DES group (n = 389) vs. BMS group (n = 480) with odds ratio: 0.68; CI: 0.45 to 1.01; P = 0.06] [38], suggesting that DES might confer a survival advantage in dialysis patients compared to BMS, 2) Dialysis patients treated with DES had lower risks of 1-year mortality compared with those treated with BMS. The DES group also had lower overall mortality rates. These data implied that dialysis patients treated with DES may have long-term survival benefits compared to those treated with BMS.

It has been previously reported that dialysis patients treated with revascularization had a survival advantage over those who are not [39, 40]. Although SYNTAX trial demonstrated the superiority of surgical revascularization in patients with complex coronary lesions, like many other cardiovascular trials, data from dialysis patients were still not available [41, 42]. However, higher risk of peri-operative complications and higher short- and medium-term mortality in dialysis patients weaken the potential long-term survival benefit of CABG [43–45]. Charytan et al. reported that CABG operative mortality for patients with ESRD was greater than 3-fold that of patients with normal kidney function. The risk of perioperative stroke was doubled in these patients, and the risk of perioperative infection was also increased significantly [43]. It is therefore important to further study the issue of PCI treatment with DES or BMS implantation.

The strengths of this study include the use of electronic records from a national health insurance registry and a uniform approach to outcome ascertainment. However, this study has some limitations inherent in a health insurance database. First, the NHI data do not include information about the severity and complexity of disease, such as lesions involving left main coronary artery, chronic total occlusion, bifurcation lesions, and the SYNTAX score. Second, they also do not include information about procedural characteristics such as total stent length, stent diameter, and whether there was a complete revascularization or not. It is possible that the missing information might have affected the outcome. Third, there were no data about follow-up angiographic results and restenosis. Therefore, strictly randomized controlled trials would help clarify the efficacy of DES.

In conclusion, this cohort study identified some survival advantages of DES over BMS implantation for dialysis patients. Both 1-year and overall composite MACE and mortality were significantly higher in the BMS group. However, it is important to validate this hypothesis in a larger, randomized trial with long-term follow-up.
